# Impact of COVID-19 on lifestyles related etiquette among nursing staff in India: a cross sectional descriptive e-survey*

**DOI:** 10.17533/udea.iee.v41n1e06

**Published:** 2023-03-14

**Authors:** Nipin Kalal, Suresh K Sharma, Nimarta Rana, Ashok Kumar

**Affiliations:** 1 Assistant Professor. College of Nursing, All India Institute of Medical Sciences, Jodhpur, Raj, India. Email: kalalnipin@gmail.com.. All India Institute Of Medical Sciences College of Nursing All India Institute of Medical Sciences Jodhpur Raj India kalalnipin@gmail.com; 2 Ph.D. Professor. College of Nursing, All India Institute of Medical Sciences, Jodhpur, Raj, India. Email: id-sk.aiims17@gmail.com All India Institute Of Medical Sciences College of Nursing All India Institute of Medical Sciences Jodhpur Raj India id-sk.aiims17@gmail.com; 3 Assistant Professor, College of Nursing, All India Institute of Medical Sciences, Jodhpur, Raj, India. Email: nimartarana@gmail.com All India Institute Of Medical Sciences College of Nursing All India Institute of Medical Sciences Jodhpur Raj India nimartarana@gmail.com; 4 Ph.D. Associate Professor. College of Nursing, All India Institute of Medical Sciences, Jodhpur, Raj, India. Email: ashokbishnoi11@gmail.com All India Institute Of Medical Sciences College of Nursing All India Institute of Medical Sciences Jodhpur Raj India ashokbishnoi11@gmail.com

**Keywords:** COVID-19, healthy lifestyle, nursing staff, feeding behavior, exercise, sleep quality, COVID-19, estilo de vida saludable, personal de enfermería, conducta alimentaria, ejercicio físico, calidad de sueño, calidad del sueño, COVID-19, estilo de vida saudável, recursos humanos de enfermagem, comportamento alimentar, exercício físico, qualidade do sono

## Abstract

**Objective.:**

To analyze the impact of COVID-19 pandemics on lifestyle-related etiquettes like eating, physical activity, and sleep behavior among nursing staff in India.

**Methods.:**

A cross-sectional descriptive E-survey was conducted among 942 nursing staff. The validated electronic survey questionnaire was used to assess the changes in lifestyle-related etiquette before and during COVID-19 Pandemic.

**Results.:**

A total of 942 responses (mean age 29.01±5.7years) were collected, 53% of the respondents were men. A slight decline in healthy meal consumption pattern (*p*<0.0001) and a restriction of unhealthy food items were observed (*p*<0.0001), and also reduction in physical activity coupled with decreased participation in leisure-related activities was seen (*p*<0.0001). The stress and anxiety slightly increase during COVID-19 pandemics (*p*<0.0001). Additionally, social support extended by family and friends to maintain healthy lifestyle-related behaviors also significantly decreased during COVID-19 PANDEMIC pandemics compared to before (*p*<0.0001). Although the COVID-19 Pandemic slightly reduced the intake of healthy meals and deterred participants from consuming unhealthy food, this this may have led to individual weight loss.

**Conclusion.:**

In general, there was a negative impact on, lifestyle like diet, sleep and mental health was observed. A detailed understanding of these factors can help to develop interventions to mitigate the harmful lifestyle-related etiquette that has manifested during COVID-19 Pandemic.

## Resumen

**Objetivo.:**

Analizar el impacto de la pandemia por COVID-19 en las etiquetas relacionadas con el estilo de vida (alimentación, actividad física y comportamiento del sueño) entre enfermeras de la India.

**Método.:**

Se realizó un estudio de corte transversal en el que participaron 942 enfermeros de todo el país. Se utilizó un cuestionario electrónico validado para evaluar los cambios en los estilos de vida antes y durante la pandemia por COVID-19.

**Resultados.:**

La edad media fue de 29.01±5.7años, 53% fueron hombres. En comparación con antes de la pandemia por COVID-19, durante la misma se observó un ligero descenso en el patrón de consumo de comidas saludables (*p*<0.0001) y se observó una restricción de alimentos poco saludables (*p*<0.0001, además de una reducción de la actividad física junto con una menor participación en actividades relacionadas con el ocio (*p*<0.0001). El estrés y la ansiedad aumentaron ligeramente (*p*<0.0001). Además, el apoyo social prestado por la familia y los amigos para mantener comportamientos saludables relacionados con el estilo de vida también disminuyó significativamente durante la pandemia en comparación a antes (*p*<0.0001). Aunque la pandemia por COVID-19 redujo ligeramente la ingesta de comidas saludables y disuadió a los participantes de consumir alimentos poco saludables, esto puede haber conducido a una pérdida de peso individual.

**Conclusión.:**

En general, se observó un impacto negativo en el estilo de vida, como la dieta, el sueño y la salud mental. Una comprensión detallada de estos factores puede ayudar a desarrollar intervenciones para mitigar la etiqueta nociva relacionada con el estilo de vida que se ha manifestado durante la pandemia COVID-19 en los enfermeros en India.

**Objetivo.:**

Analisar o impacto da pandemia de COVID-19 nos rótulos do estilo de vida (dieta, atividade física e comportamento do sono) entre enfermeiras na Índia.

**Método.:**

Foi realizado um estudo transversal do qual participaram 942 enfermeiros de todo o país. Um questionário eletrônico validado foi usado para avaliar as mudanças no estilo de vida antes e durante a pandemia de COVID-19.

**Resultados.:**

A média de idade foi de 29.01±5.7 anos, 53% eram homens. Comparado com antes da pandemia de COVID-19, observou-se uma leve diminuição no padrão de consumo de refeições saudáveis durante a pandemia (*p*<0.0001), além de redução da atividade física e menor participação em atividades relacionadas ao lazer (*p*<0.0001). O estresse e a ansiedade aumentaram ligeiramente (*p*<0.0001). Além disso, o apoio social fornecido pela família e amigos para manter comportamentos de estilo de vida saudável também diminuiu significativamente durante a pandemia em comparação com antes dela (*p*<0.0001).

**Conclusão.:**

Em geral, foi observado um impacto negativo no estilo de vida, como dieta, sono e saúde mental. Uma compreensão detalhada desses fatores pode ajudar a desenvolver intervenções para mitigar a etiqueta prejudicial relacionada ao estilo de vida que se manifestou durante a pandemia de COVID-19 entre enfermeiras na Índia.

## Introduction

COVID-19 PANDEMIC has not only changes the entire routine of an individuals, but lifestyle is also changed. General public has changed their routine lifestyle to boost immunity and fight infection. Several factors compound overtime to completely change lifestyle-related behaviors, especially daily eating, physical activity and, sleep behaviors that are known to be independent risk factors for metabolic complications such as, diabetes obesity and cardiovascular disorders.^1^^,^^2^

Unhealthy lifestyle habits such as poor diet, lack of physical activity, smoking, and alcohol consumption; not only contribute to the global burden of disease^3^ but they are also associated with poor mental health outcomes.^4^ During the COVID-19 pandemic outbreak, lifestyle guidelines have recently emphasised maintaining a healthy nutritional status and engaging in physical activity at home.^5^^,^^6^ Social media and government also emphases regarding the adaptation of a healthy lifestyle to protect from infectious disease. During lockdown, nursing staff routine has changed due to long duty hours and changing of shift duty hours. As to become fit and fine, they also focusing on health lifestyle. It has been shown that people who are stressed seek more high-fat and high-sugar foods, as their bodies require more energy to function.^7^ Furthermore, the body stores more abdominal fat.^8^ It is well known that negative emotions can contribute to overeating, known as "emotional eating”.^9^

At present, there are only few studies reporting on the well-being impacts of COVID-19 PANDEMIC, which is confirming that COVID-19 PANDEMIC may be having widespread impacts on a range of health behaviors and, ultimately, mental health.^10^ Stress has also been implicated in poor sleep quality and disrupted sleep.^11^ The COVID-19 PANDEMIC pandemic has been associated with poor sleep quality in China^12^ and Italy, with over half of Italian respondents experiencing poor sleep quality.^13^ Limited studies displayed the impact of COVID-19 Pandemic on lifestyle changes among the general public but not any of the studies is related to changing of lifestyle-related etiquette during COVID-19 Pandemic among nursing staff. As they are the frontline warriors, so it is essential to know the key points of how their lifestyle during COVID-19 PANDEMIC is affected, what kind of changes are made to fight this virus, what are the reason to change this lifestyle-related etiquette. It has been hypothesized that the increase in unstructured time and the psychological impact resulting from the enforced quarantine might induce changes in dietary habits and lifestyle. There is a lack of evidence that evaluates the lifestyle-related etiquette among nursing staff of India. Hence, the current was conducted to know the impact of COVID-19 PANDEMIC on lifestyles related etiquette among nursing staff in India.

## Methods

Study design. We conducted a cross-sectional descriptive E-survey among nursing staff in India. The study was carried out in the months of May and June 2021. 

Study setting and study subjects. We have included all the categories of nursing staff (Deputy Nursing Superintendent, Assistant Nursing Superintendent, Senior Nursing Officer Nursing officers) from across the country. The sample size was calculated with Z1−α/2 = Critical value and a standard value for the corresponding level of confidence (At 95% CI or 5% level of significance (type-I error) it is 1.96 and at 99% CI it is 2.58). On the basis of a study done by Chopra *et.al*^14^ considering P =0.29; q=0.71; d=0.03). The sample size was calculated as 858 while considering a 10% non-response rate, a total sample size of 942 participants was considered for this present study.

Data collection, and questionnaire validation. A questionnaire was shared via a google form, where a brief description of the study with its objectives was mentioned. It is also declared that the confidentiality and anonymity of the subjects and the data collected will be maintained throughout the study. All the participants were enrolled in the study after giving informed consent in a google form, then only they can proceed to the next step. A professional invitation along with a link was shared among all personal and professional group members via WhatsApp, Facebook, and email to facilitate the data collection.

We asked all known faculties of the entire country to circulate this survey national wide. We also requested all nursing staff to share this link with friends and all known nursing colleagues to get a maximum number of participants in the pandemic situation. The researcher followed up on the status of data collection a month after the survey link was delivered in all the states. The electronic survey questionnaire was designed to assess changes in multiple lifestyle-related etiquettes such as eating, physical activity, sleep, and other health-related behaviors during the COVID-19 PANDEMIC outbreak. The differential questionnaire used in this study was developed and validated as an extension of a short version lifestyle-related practices questionnaire in Indian adults.^15^

The questionnaire consisted of three sections, where section one consists of the socio-demographic data, the infection status of COVID-19 PANDEMIC, self-reported anthropometric data, and one question on change in weight status during COVID-19 pandemic, etc. Section second consists of two parts with 22 items in each and it assessed the changes in lifestyle-related etiquette. Part A (A1 to A22) assesses the baseline lifestyle-related behaviors and Part B (B1 to B22) evaluates changes in different lifestyle-related behaviors such as eating habits, physical activity, and sleep patterns during the pandemic. The domain of eating behavior consists of 11 items on meal pattern, portion size, frequency of meals, food group consumption pattern, emotional eating, and intake of high fat, salt, and sugar (HFSS) foods and sugar, sweetened beverages (SSB) consumption. The domain of physical activity pattern has five items focusing on different components of activity such as aerobic exercise, involvement in household chores, leisure-related activity, and sitting time. Two items are for the assessment of sleep patterns, one item for daily stress levels, and two items for stress-related addictive behaviors such as smoking and alcohol consumption. The five-point Likert-response choices are as follows: ‘not at all, ‘rarely’, ‘sometimes’, ‘most of the days’, and daily’. The magnitude of the response’s ranges from 5 (most acceptable behavior) to 1 (least acceptable behavior). The third section has six items, intended to measure the COVID-19 pandemic specific reasons for the changes in these behaviors. 

Data analysis. Data were coded and entered into an excel sheet and Statistical Package for the Social Sciences (SPSS 27) for statistical analysis. Descriptive were used as demographics of the participants as frequency and, percentage. The responses for before COVID-19 pandemic lifestyle scores and during-COVID-19 pandemic lifestyle scores were assessed and these scores were subtracted for each item giving the mean difference scores which were associated with demographic variables. The association between the categorical variables was assessed using the independent t-test and, ANOVA test, and *p*<0.05 was considered statistically significant.

Ethical considerations. Ethical clearance was obtained from the Institutional Ethics Committee-wide letter no. AIIMS/IEC/2021/3623.

## Results

Table 1 depicts the demographic details of the participants (*n*=942). Of all the participants, 499 (53%) were male with the mean age of 29.01±5.74. Out of all, 315 (33.4%) participants, and 317 (33.7%) their family members had a history of COVID-19 infection. Less than one- fourth 209 (22.2%) participants reported a weight gain during the COVID-19 PANDEMIC pandemic and, 303 (32.2%) reported weight loss. The mean self-reported body mass index (BMI) was 23.70±4.08kg/m^2^. 

**Table 1:** Socio-demographic characteristics of the 942 participants

Variables	Frequency
Age in year; mean ± SD	29.01±5.74
**Gender; *n* (%)**	
Male	499 (53)
Female	443 (43)
**Education qualification; *n* (%)**	
GNM	116 (12.3)
PB B.Sc.	71 (7.5)
B.Sc. Nursing	609 (64.6)
M.Sc. Nursing	146 (15.5)
**Place of living; *n* (%)**	
Rural	198 (21)
Urban	744 (79)
**Marital status; *n* (%)**	
Married	553 (58.7)
Unmarried	383 (40.7)
Divorce/widow/widower	06 (0.6)
**Did you ever had history of COVID-19 infection? ; *n* (%)**	
Yes	315 (33.4)
No	627 (66.6)
**Did any of your family member had COVID-19 infection? ; *n* (%)**	
Yes	317 (33.7)
No	625 (66.3)
**Employment under; *n* (%)**	
Central Government	627 (66.6)
State Government	133 (14.1)
Private organization/Hospital	182 (19.3)
**Weight gain during COVID-19 PANDEMIC; *n* (%)**	
Yes	209 (22.2)
No	733 (77.81)
**Weight loss during COVID-19 PANDEMIC; *n* (%)**	
Yes	303 (32.2)
No	639 (67.83)
Self-reported BMI; mean ± SD	23.70±4.08

* Independent t test of ANOVA

Table 2 depicts the comparison of mean scores of lifestyle-related behaviors before and during COVID-19 pandemic. There was a significant decrease in routine consumption of meals at regular intervals during COVID-19 Pandemic. The intake of unhealthy food items such as fast food, junk food, was significantly declined during COVID-19 pandemic. Moreover, the participation in moderate intensity aerobic exercises, participation in household chores, participation in leisure related activities was declined significantly. With regard to sleeping pattern, the quality of sleep was declined during pandemic. In terms of other health-related behaviors such as level of stress or anxiety and alcohol consumption, is slightly increased during Covid- 19 pandemic.With concern to social support extended by family and friends to maintain healthy lifestyle-related behaviors also significantly decreased.

**Table 2:** Comparison of mean lifestyle-related etiquette score before and during COVID-19 of participants

Lifestyle-related etiquette	COVID-19 PANDEMIC	Pandemics	Mean difference (Mean ± SD)	Paired t-test (*p*-value)
	Before Mean ± SD	During Mean ± SD		
Consumption of regular meal pattern	4.07±1.11	3.71±1.25	-0.36±1.03	<0.0001
Consumption of fast food	3.62±0.96	4.26±0.90	0.64±0.92	<0.0001
Consumption of junk foods as snacks	3.78±0.93	4.27±0.90	0.49±0.88	<0.0001
Frequency of your fruits and vegetables intake	3.75±0.88	3.76±0.99	0.01±0.90	0.667
Consumption of balanced diet by including healthy ingredients	3.84±0.90	3.79±0.98	-0.05±0.76	0.056
Consumption of milk or its products	3.83±0.94	3.72±1.09	-0.11±0.85	<0.0001
Consumption of one or more servings of pulses, egg or meat in a day	3.06±1.17	2.97±1.25	-0.09±0.71	<0.0001
Daily Consumption of sugar/honey/jiggery	2.85±1.18	2.97±1.25	0.12±0.74	<0.0001
Consumption of sugar sweetened beverages	3.48±1.04	3.74±1.06	0.26±0.73	<0.0001
Consumption of foods with high sugar	3.84±0.91	4.03±0.89	0.19±0.63	<0.0001
Emotional Eating	4.08±0.91	3.71±1.13	-0.37±0.87	<0.0001
Participation in 30 min of moderate intensity aerobic exercises/sports	3.02±1.20	2.75±1.21	-0.27±1.10	<0.0001
Participation in household chores	3.50±1.18	3.29±1.26	-0.21±1.10	<0.0001
Participation in leisure related activities	3.38±0.99	2.42±1.15	-0.96±1.19	<0.0001
Daily sitting time at work	3.35±1.29	3.53±1.43	0.18±1.15	<0.0001
Breaks from sitting	3.13±1.15	3.28±1.31	0.15±1.01	<0.0001
Daily hours of sleep	1.90±0.48	1.95±0.65	0.05±0.70	0.017
Quality of sleep	3.81±0.83	3.20±0.94	-0.61±1.05	<0.0001
Level of stress or anxiety	3.15±1.19	4.16±0.79	-1.01±1.13	<0.0001
Smoking	4.92±0.39	4.93±0.37	0.01±0.16	0.050
Alcohol Consumption	4.78±0.56	4.83±0.53	0.05±0.34	<0.0001
Social support	3.99±1.16	3.63±1.37	-0.36±1.13	<0.0001

The association of comparing mean difference of during and before COVID-19 PANDEMIC lifestyle-related behavior scores with socio-demographic characteristics of participants. With regard to gender category there is mean difference between males and females concerning to eating behavior (*p*<0.0001). In terms of place of living category, there is no mean difference between rural and urban area with respect to eating behavior (*p*=0.150) physical activity (*p*=0.397), sleep pattern (*p*=0.227) and other behavior (*p=0*.479). However, with regard to the marital status, there is only significant difference in physical activity (p=0.038) and other behavior (*p*=0.038). (Table 3).

**Table 3:** Comparing mean difference of during and before COVID-19 PANDEMIC lifestyle-related behavior score with socio-demographic characteristics of participants

Characteristic difference between scores	Eating Behavior Mean±SD	p-value	Physical Activity Behavior Mean±SD	***p-*value**	Sleep Pattern	***p*-value**	Other Behavior Mean±SD	***p*-value**
Gender								
Male	0.01±0.36	<0.0001*	0.23±0.55	0.265*	0.61±0.25	0.201*	0.057±0.14	0.265*
Female	-0.09±0.35		0.22±0.51		0.72±0.24		0.06±0.13	
Place of living								
Rural	-0.07 ± 0.33	0.150*	0.25±0.55	0.397*	0.68±0.23	0.227*	0.07±0.14	0.479*
Urban	-0.03±0.37		0.22±0.54		0.72±0.25		0.06±0.14	
Marital Status								
Married	-0.02± 0.36	0.059	0.26±0.57	0.038	0.71±0.26	0.422	0.07±0.15	0.038
Unmarried	-0.07±0.36		0.18±0.50		0.73±0.23		0.05±0.13	
Others	0.07± 0.17		0.10±0.21		0.71±0.12		0.03±0.06	
Education Qualification								
GNM	-0.05± 0.32	0.438	0.20±0.49	0.575	0.75±0.23	0.388	0.06±0.13	0.575
PB B.Sc.	-0.01± 0.34		0.22±0.48		0.72±0.23		0.06±0.12	
B.Sc. Nursing	-0.03± 0.37		0.24±0.56		0.71±0.25		0.6±0.14	
M.Sc. Nursing	-0.08± 0.35		0.18±0.54		0.71±0.24		0.05±0.14	

* Independent t test, ANOVA

Study reported that the main reason for lifestyle changes during COVID-19 Pandemic were worrying about family and friends (64.86%) followed by stress and anxiety (Table 4).

**Table 4:** Frequency of participant responses for lifestyle changes during COVID-19 pandemic

Reasons for lifestyle changes during COVID-19 pandemic	Frequency (%)
Lack of access to fresh fruits and vegetables	58.50
Fear of coronavirus spread through food	41.62
Activity adopted as Yoga	38.44
Social restrictions to parks and public places	42.99
Stress and anxiety	41.94
Worrying about family and friends	64.86

## Discussion

The outbreak of COVID-19 PANDEMIC and measures of its containment has an evident impact on the lifestyle-related behaviors in the population. In the present study we assessed the impact of COVID-19 PANDEMIC on lifestyle-related etiquette. The mean age of the patients who participated in the current study was 29.01±5.74 whereas the study by Chopra S et al which reported mean age was 33.33±14.5.^14^ The findings of our study revealed that the habit of consuming meals routinely at regular intervals has slightly decreased during COVID-19 pandemic however the finding by Chopra *et al.*, where slight improvement in routine consumption of meals at regular intervals.^14^

The results of the present study the reported that participant’s consumption of daily fruits and vegetables intake, balanced diet, is not significantly increased, these findings are not corresponding to studies performed by Di Renzo where they reported that 37.4% of the study population declares to eat more (fruit, vegetables, nuts and legumes).^16^ The present study demonstrates that the consumption of sugar sweetened beverages, foods with high sugar is significantly increased. Scarmozzino in his study also presented similar findings that participant’s consumption of sweet foods is increased (42.5%).^17^ It is found that in the present study, the participants refraining from alcohol (89.2%) was increased during the pandemic was similar to the study done by Di Renzo and Scarmozzino, which reported a decrease of the intake of alcohol.^16^^,^^17^

Results of our study demonstrated that, a decrease in daily participation of moderate- intensity exercises from (12% to 9.2%), was in loop with another study by Radwan H, which also reported, decreased physical activity (30%).^18^ It is identified that participants reported a weight gain during COVID-19 PANDEMIC, these findings are comparable with studies done in Italy, USA, Spain, UAE.^17^^,^^19^^-^^20^ The findings of our study revealed that social support extended by family and friends to maintain healthy lifestyle-related behaviors also significantly decreased. The finding of our study are in the loop with studies conducted by Balanzá-Martínez where they reported that no found significant changes in social support however as per another study conducted by Zhang reported that the majority of participants reported that they received increased support from friends and increased support from family members.^21^^,^^22^

Additionally, the current study findings suggested that there is no statistically significant difference between physical activity of males and females, these findings are corresponding to studies performed by Chopra S where they reported overall physical activity worsened in both the genders, but men experienced a greater significant reduction in their activity status in comparison to females. The results of our study illustrated that the reason for lifestyle changes during COVID-19 Pandemic pandemic is stress/ anxiety (41.94%), these findings are consistent with the findings of Scarmozzino F et al study, which reported (42.7%) of participant’s higher anxiety levels.^14^^,^^17^

## Conclusion

The findings of this study are consistent with western literature in demonstrating that one possible effect of the COVID-19 pandemic is adaption of harmful lifestyle-related behaviours to prevent coronavirus infection. The study's findings suggest that the preventive measures taken to control the coronavirus have a mixed effect on lifestyle-related behaviour,

Recommendations. The primary strength of the current study, is answer to a demanding question at an unprecedented time and in fact the survey was conducted quickly in the most critical period of the pandemic in India, less than three weeks after the lockdown. Furthermore, the study was prospective, with a clearly defined time point well before and definitively during COVID-19 Pandemic and was conducted in nursing staff, while other studies have been retrospective and done in general population. Likewise, the research may provide some guidelines for larger surveys in the future. It is recommended that large-scale studies with both qualitative and quantitative methods should be conducted in all regions of India, particularly most severely impacted by the pandemic. 

Limitations. First, the present study included the non-probabilistic nature of the sample which precludes generalizability of the results to the entire population. Second, perceived lifestyle changes were self-reported by participants thus these measurements may be predisposed to recall bias. Third, authors are not able to get equivalent number of participants from all the states. Also, as the study was conducted in second wave of COVID-19 PANDEMIC, so may be nursing staff are adopted to new lifestyle etiquettes. New technologies, such as personal health trackers, may be useful to overcome these limitations in the future, but under confinement these are difficult to implement.
